# Proximal fragment perfusion following hip arthroplasty with subtrochanteric shortening osteotomy in cases with severe developmental dysplasia of the hip

**DOI:** 10.55730/1300-0144.6144

**Published:** 2025-10-26

**Authors:** Vedat BİÇİCİ, Hilmi ALKAN, Enejd VEİZİ, Şahan GÜVEN, Serkan ÜNLÜ, Elif ÖZDEMİR, Tural TALIBLI, Ahmet FIRAT

**Affiliations:** 1 Department of Orthopedics and Traumatology, Ankara Etlik City Hospital, Ankara, Turkiye; 2 Department of Orthopedics and Traumatology, Ankara Bilkent City Hospital, Ankara, Turkiye; 3 Department of Nuclear Medicine, Ankara Bilkent City Hospital, Ankara, Turkiye; 4 Department of Orthopedics and Traumatology, VM MedikalPark Ankara Hospital, Ankara, Turkiye

**Keywords:** Hip arthroplasty, subtrochanteric osteotomy, perfusion, bone scintigraphy

## Abstract

**Background/aim:**

The changes in proximal femoral blood flow occurring after reconstructive surgery for Crowe types III–IV developmental dysplasia of the hip (DDH) and their impact on healing remain unclear. In the present study, blood flow around the osteotomy site is evaluated with particular focus on the proximal segment in patients who underwent subtrochanteric shortening osteotomy and hip arthroplasty. To this end, single-photon emission computed tomography (SPECT/CT) scans were conducted to evaluate changes in proximal blood flow and to assess the impact on union rates and potential complications.

**Materials and methods:**

A retrospective analysis was conducted of 26 hips from a total of 20 patients with Crowe types III or IV DDH who underwent hip arthroplasty with subtrochanteric shortening osteotomy between July 2017 and September 2022. Planar, whole-body, and SPECT/CT images were reviewed by two nuclear medicine physicians, and the tracer uptake between the greater and lesser trochanters was assessed visually and quantitatively.

**Results:**

Assessed in the study were 26 hips of 20 patients (mean age 51.5 ± 8.8 years; 80.8% female) with a mean follow-up of 20.1 ± 10.1 months. Of the total, 14 hips were right-sided and 12 were left-sided; five and 21 were Crowe types III and IV, respectively. The union was timely (≤6 months) in 16 hips (61.5%), delayed in nine hips (34.6%), and nonunion in one hip (3.8%). After one year, 25 hips (96.2%) had achieved union. The mean femoral shortening was 3.8 ± 1.2 cm. SPECT/CT analysis revealed a mean SUV_mean_ of 3.5 ± 1.2 on the operated side versus 3.3 ± 1.2 on the contralateral side (p = 0.350). The tracer uptake ratios for greater trochanter/sacrum and greater trochanter/distal femur were similar between sides (p = 0.425 and 0.674, respectively).

**Conclusion:**

In patients with Crowe types III–IV DDH undergoing total hip arthroplasty, shortening osteotomies and soft tissue releases do not appear to significantly reduce vascularity or perfusion of the proximal osteotomy fragment. In our cohort, high union rates were observed.

## 1. Introduction

Reconstructive treatments for Crowe type III and IV hip dislocations in developmental dysplasia of the hip (DDH) are among the most challenging hip surgeries in orthopedics [1]. Such high-grade dislocations, characterized by anatomical abnormalities, biomechanical alterations, femoral deformities, and severe soft tissue contractures, increase the complexity of total hip arthroplasty (THA) [2,3]. Anatomical reconstruction of the hip rotation center is considered the optimum treatment approach in cases with high dislocations [4]. However, restoring the anatomical hip center often requires limb lengthening that may exceed 4 cm, potentially placing excessive stress on neurovascular structures and complicating hip reduction.

To overcome these difficulties, subtrochanteric shortening osteotomy (SSO) is performed in conjunction with THA [3,5,6]. True anatomical reconstruction and proper restoration of the hip rotation center in the true acetabulum require the pathological changes in the soft tissues caused by DDH to be addressed, involving the excision of the thickened and contracted capsule, fibrotic tissues, and osteophytes. Additionally, the psoas tendon is released, and periarticular muscles are stripped from their femoral attachments. These interventions are essential not only to facilitate reduction, but also to prevent residual soft tissue contractures that may lead to gait abnormalities postoperatively. During such procedures, often only the gluteus medius attachment is left intact, while almost all soft tissues proximal to the osteotomy site are released from their femoral attachments [7,8]. Successful surgery and the patient’s ability to bear weight and walk properly postoperatively depend on both prosthesis-bone integration and osteotomy healing.

During such challenging procedures, orthopedic surgeons must keep in mind the potential reduced blood supply to the proximal fragment of the osteotomy site due to the associated risk of secondary complications, including osteonecrosis and delayed union, especially after extensive soft tissue releases [9]. The changes in proximal femoral blood flow after surgery and their impact on healing are not well understood. Single-photon emission computed tomography (SPECT/CT), which combines functional radionuclide imaging with high-resolution structural computed tomography (CT), is most commonly used to detect the spread of metastatic cancer [10]. Since SPECT/CT provides both structural and functional information, it has been utilized not only for the diagnosis and follow-up of bone metastases, but also in orthopedics for evaluating bone graft and bone viability, osteomyelitis, osteointegration, and early implant loosening [10–13].

In postoperative X-ray imaging, assessing the viability of the proximal femur after soft tissue releases and osteotomies is not always possible. Consequently, the impact of potentially nonviable areas on postoperative healing remains unclear. Despite the widespread use of SSO in combination with THA for Crowe type III–IV DDH, there is a notable lack of studies analyzing changes in proximal femoral blood flow and bone perfusion using such functional imaging modalities as SPECT/CT following these procedures. Most previous studies have focused on clinical outcomes, radiographic alignment, or prosthesis integration, while the potential effects of extensive soft tissue release and osteotomy on local vascularity and postoperative healing remain poorly understood. Understanding these perfusion changes is crucial as they may influence osteotomy union, increase the risk of delayed or nonunion, and hamper overall postoperative recovery. The present study aims to fill this gap by investigating proximal femoral perfusion and bone viability using bone SPECT/CT, providing objective insights into the biological impact of SSO and associated soft tissue releases. Given the limited studies reporting functional imaging after SSO, we hypothesize that proximal femoral blood flow and bone viability are preserved, despite extensive soft tissue releases, in patients with Crowe types III–IV DDH undergoing THA. To this end, the present study analyzes changes in proximal femoral blood flow and evaluates bone viability using SPECT/CT in patients who underwent hip arthroplasty with subtrochanteric shortening osteotomy. The study goes on to discuss the implications of these changes for postoperative recovery.

## 2. Materials and methods

### 2.1. Patient selection

Institutional Ethics Committee approval was obtained for the study (decision No. TADEB 1-24-822), all patients provided verbal and written consent for the use of their data, and the study was conducted in accordance with principles laid out in the Declaration of Helsinki. This is a retrospective cohort study of patients who underwent total hip arthroplasty and postoperative SPECT/CT imaging at our institution between July 2017 and September 2023. Postoperative SPECT/CT imaging of the pelvis was routinely performed in the sixth postoperative month during the study period, rather than conventional CT, to assess osteotomy healing and final implant position. The combination of SPECT and low-dose CT ensured that total radiation exposure did not exceed 9 mSv, resulting in lower radiation exposure than routine CT scans [14].

Included in the study were patients with Crowe type III or IV DDH, according to the Crowe classification, who provided informed consent for surgery, and who had a minimum of 12 months of radiological follow-up.

Excluded from the study were patients with asymptomatic high hip subluxations, prior hip surgeries (e.g., osteotomy or trauma), inflammatory or oncological diseases affecting the operated hip joint region, neuromuscular impairments in the symptomatic extremity, and those with incomplete follow-up.

Subsequently, a total of 26 hips belonging to 20 patients diagnosed with Crowe type III or IV developmental dysplasia who underwent primary cementless total hip arthroplasty combined with transverse subtrochanteric shortening osteotomy were included in the study.

### 2.2. Surgical technique

All procedures were performed by the same experienced surgeon (or surgical team) to minimize variability. Surgeries were conducted with the patient in the lateral decubitus position using a posterolateral approach to the hip. A long incision was made to access the proximal femur and pseudo-acetabulum. The vastus lateralis was elevated superiorly, and the gluteus maximus tendon and external rotators were incised. The psoas tendon was then released, and all posterior femoral structures were loosened. A femoral osteotomy was performed distal to the lesser trochanter. The proximal femur above the osteotomy line was grasped with a bone clamp, and all soft tissues other than the gluteus medius and minimus were released and rotated 180 degrees superiorly (Turn Up–Turn Down technique). The femoral neck was then resected. The superior capsule was excised without damaging the gluteus minimus muscle, after which the proximal femur was retracted superiorly, and the distal femur anteriorly to expose the true acetabulum. The hypertrophic capsule and all remaining soft tissues were fully resected to allow access to the true acetabulum. The ischium, anterior wall, and transverse acetabular ligament were identified, and after adequately exposing the acetabulum, reaming was initiated using a 32-mm reamer and progressively continued until the medial wall of the true acetabulum was reached, as described by Sener et al. [15]. The true acetabular fossa was then deepened posteriorly and medially using the reamers. Medialization was performed to improve acetabular cup coverage, and the acetabulum was then reconstructed using an acetabular prosthesis.

After implantation of the acetabular component, the proximal femur was reamed using straight reamers, and sequential rasping was performed until the appropriate stem size was achieved. The final reamer was inserted into the proximal femur, and reaming was completed to achieve 10–15° femoral anteversion. Following hip reduction, the osteotomy line was approximated, and the estimated amount of shortening was determined.

After completing the osteotomy, a cylindrical, straight femoral stem with a porous coating and longitudinal flanges (Wagner Cone, Zimmer, Warsaw, IN) was implanted in all hips. After prosthesis implantation, the resected femoral segment was split into two pieces and secured to the osteotomy site using two or three cables. Any gaps between the osteotomy sites were filled by compacting autograft obtained from the resected cancellous bone.

### 2.3. Postoperative rehabilitation and radiographical healing

Isometric exercises and active limb training were initiated immediately after surgery. Patients were encouraged to begin early mobilization using two crutches and were allowed to bear weight in the first postoperative week. Active-assisted range of motion exercises were initiated during the second postoperative week and full weight bearing was allowed after the third or fourth week, depending on patient compliance.

Routine two-plane X-ray views were obtained from patients during every outpatient visit with union being defined as a painless continuous bridging callus or cortical continuity across at least three cortexes of the osteotomy sites within the first 6 months. Delays in union past the sixth postoperative month were defined as delayed union.

### 2.4. Technetium 99m-methyl diphosphonate (99mTc-MDP) bone scintigraphy protocol

At the sixth postoperative month, technetium 99m-methyl diphosphonate (Tc99m MDP) bone scintigraphy was performed using an integrated SPECT/CT dual-head gamma camera (GE Discovery NM/CT 670; GE Healthcare, Milwaukee, WI). Following the administration of 20 mCi 99mTc-MDP, dynamic imaging and planar blood pool imaging were performed with the entire femoral prosthesis area in the field of view. At 3 hours post-injection, whole-body imaging, planar imaging of the femoral prosthesis region, and SPECT/CT imaging were performed. Planar and SPECT imaging were acquired using a low-energy high-resolution (LEHR) parallel collimator at an energy peak of 140 keV, with a 15–20% window width, at a scanning speed of 10–12 cm/min for whole-body imaging, and either for 5 minutes, or until 750,000 counts were reached, using a 256 × 256 matrix. SPECT/CT images of the femoral prosthesis area were obtained in helical computed tomography (CT) mode with the following parameters: 2.5 mAs current, 140 kV voltage, 512×512 matrix, 5 mm slice thickness, 360° rotation, six rotation angles, and step-and-shoot acquisition mode.

### 2.5. Image analysis

**Figure 1:**
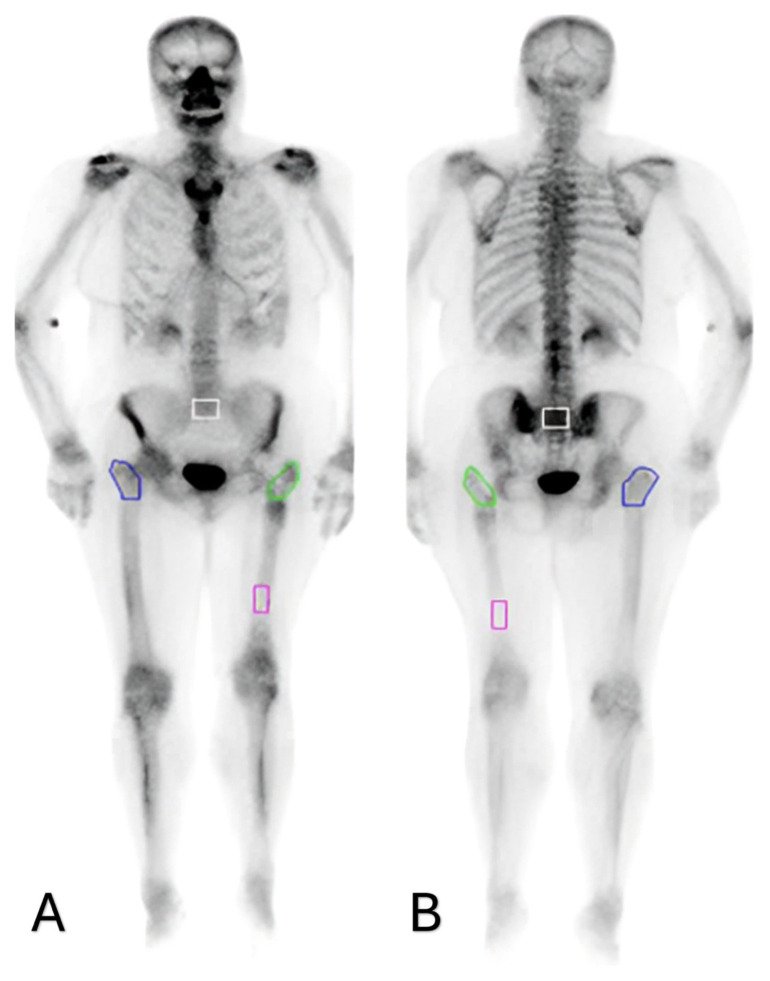
Mean counts obtained from regions of interest (ROIs) in the proximal femur, distal femur, and sacrum on planar SPECT/CT images (anterior view [A] and posterior view [B]).

**Figure 2:**
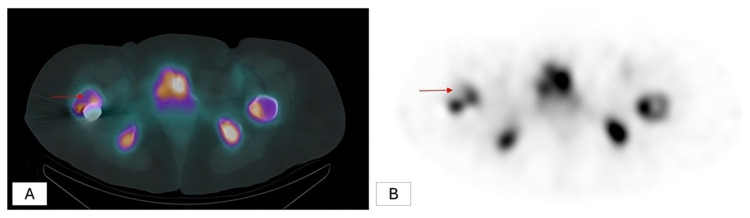
The distinction between viable and nonviable was made based on radionuclide uptake on SPECT/CT images (anterior view [A] and posterior view [B]).

Planar, whole-body, and SPECT/CT images were independently evaluated by two expert nuclear medicine physicians, and the mean values of their measurements were incorporated into the final analysis. Tracer uptake in the region between the greater and lesser trochanters of the femur was assessed both visually and quantitatively from whole-body images. The visual classification of the proximal femoral region as hypoactive, normoactive, or hyperactive was performed independently by an experienced nuclear medicine physician based on the intensity of radiotracer uptake. For the quantitative analysis, irregular regions of interest (ROIs) were defined on planar images, including the area between the greater and lesser trochanters of the operated femur, the corresponding area on the contralateral femur, the distal segment of the femoral shaft distant from the surgical site, and the sacrum. The mean counts for each ROI were obtained from both anterior and posterior views, and the geometric mean values were recorded (Figure 1A and 1B). Areas showing no radionuclide uptake on SPECT/CT images were classified as nonviable (Figure 2A and 2B).

**Figure 3:**
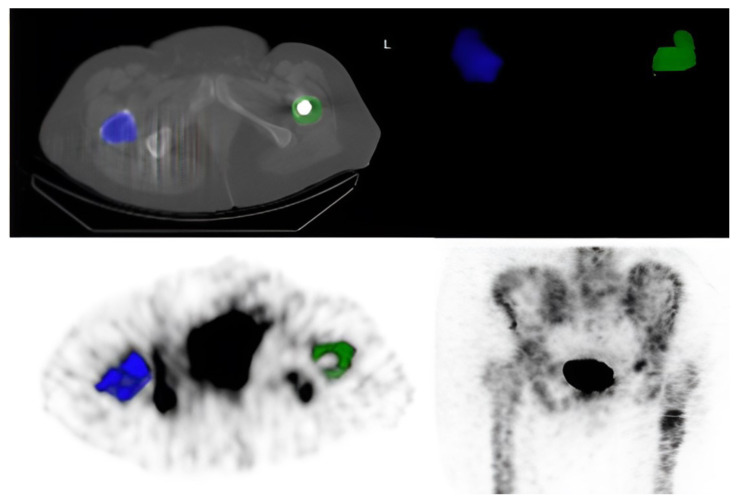
Image comparing radionuclide uptake between the greater and lesser trochanters of the femur on the operated and nonoperated sides.

Using Q.Metrix software in a GE AW Volume Share 7 workstation, volumes of interest (VOIs) were defined between the greater and lesser trochanters of the operated femur, and radionuclide uptake was quantified based on mean standardized uptake values (SUV_mean_) and maximum standardized uptake values (SUV_max_) (Figure 3).

The same procedure was then applied to the region between the greater and lesser trochanters of the contralateral femur. SPECT/CT images were analyzed without attenuation correction, and the volumes of nonviable areas as well as the total volume between the greater and lesser trochanters were calculated.

## 3. Statistical analysis

Statistical analyses were performed using IBM SPSS Statistics, Version 22.0 (IBM Corp., 2011). The normality of the data distribution was evaluated using visual (histograms and probability plots) and analytical (Kolmogorov–Smirnov test) methods. Descriptive statistics for numerical variables were expressed as mean + standard deviation, while categorical data were expressed as numbers and percentages. A Mann–Whitney U test was used for the statistical comparison of the operated and contralateral perfusion data. Interobserver reliability was assessed for the radiological measurements and an intraclass correlation coefficient (ICC) was calculated. A p-value of <0.05 was considered statistically significant.

## 4. Results

The mean patient age was 51.5 ± 8.8 (range 37–71) years and the majority of patients were female (80.8%). The mean follow-up was 20.1 ± 10.1 (range 12–49) months. There were 12 left and 14 right hips, six of which were bilateral (Table 1).

Unions were considered timely in 16 hips (61.5%) and delayed in nine hips (34.6%) at the sixth month follow-up. By the end of the first year, all but one osteotomy site had achieved radiographic union. Radiological data is presented in Table 2.

Of the total, eight hips were classified as normoactive, 17 as hyperactive, and one as hypoactive. The mean volume of the region between the greater and lesser trochanter was 31.5 cm^3^ (range 15–75 cm^3^), while the mean nonviable volume was 3.5 cm^3^ (range 0–16 cm^3^). The mean ratio of nonviable volume to total volume was 9.8%.

Reliability analysis demonstrated good-to-excellent inter-observer agreement (ICC = 0.893), indicating the high reproducibility of the method, consistent with previous studies using the same radiological modality [16]. In patients who underwent unilateral surgery, the mean SUV_mean_ value of the region between the greater and lesser trochanters was 3.2 ± 1.2 (range 1.7–6.2) on the operated side, compared to 3.3 (range 1.7–6.2) on the nonoperated side. The mean greater trochanter/sacrum tracer uptake ratio was 0.6 ± 0.1 (range 0.3–0.8) on the operated side and 0.5 ± 1.1 (range 0.3–0.8) on the nonoperated side. The mean greater trochanter/distal femur tracer uptake ratio was 1.4 (range 0.8–3.1) on the operated side and 1.3 (range 0.9–2.4) on the nonoperated side. None of the differences between the operated and nonoperated sides in the above comparisons were statistically significant (p = 0.350, 0.425, and 0.674, respectively) (Table 3).

Clinically, limb length equalization was achieved in the majority of patients, and no major gait abnormalities were observed at the final follow-up. Postoperative pain was minimal in most cases and improved further during the rehabilitation period.

## 5. Discussion

The most notable finding of this study is that adequate vascularization of the trochanteric region is maintained following hip arthroplasty with subtrochanteric shortening osteotomy in patients with severe developmental dysplasia of the hip. At a minimum of 1-year follow-up, the majority of patients exhibited normoactive or hyperactive characteristics on SPECT/CT, indicating complete union of the osteotomy site or ongoing bone remodeling.

Historically, the treatment of hips with Crowe type III–IV DDH has been challenging, and there remains a lack of consensus on the optimal surgical management approach. Recent studies have reported favorable clinical outcomes when the acetabular cup is placed between the true and false acetabulum without performing an osteotomy [17,18]. That said, there are also studies advocating for the lowering of the rotational center to the true acetabulum to reduce failure rates and alleviate contact load [19–21], a procedure that often requires a shortening osteotomy. Osteotomy facilitates the reduction of highly dislocated hips to the true acetabulum but necessitates extensive soft tissue release, which can compromise the vascularity of the proximal fragment [1–3,22–24]. Vascular changes in the trochanteric region after a hip arthroplasty were first noted by ElMaraghy et al. [25], who reported a decrease of up to 61% from the original baseline after implantation. In their study, intraoperative measurements were performed using a laser Doppler flowmeter, which highlighted that despite conservative soft tissue release during implantation, local blood flow could still be compromised. In contrast to ElMaraghy’s intraoperative findings, our postoperative SPECT/CT analysis demonstrated preserved perfusion in the trochanteric region at follow-up, suggesting that vascular compromise, if present initially, is transient and recovers over time. While there have been several studies to date investigating the perfusion status of the femoral neck after a hip resurfacing [26], none have investigated the perfusion of the proximal fragment after a shortening osteotomy. The results of the present study, while not recorded intraoperatively, showed that at a minimum of 1 year, the proximal fragment was well vascularized, while the remodeling process was continuing in some cases.

Nonunion, although rare, is a challenging complication after a shortening femoral osteotomy. Kawai et al. [27] reported that 81.5% patients treated with a shortening osteotomy showed signs of union after a follow-up of 12 months with only two patients requiring revision due to nonunion, while Masson et al. [28] reported a nonunion rate of 13.8%. Our results largely concur with these findings, with one nonunion (3.8%) and a delayed union rate of 34.6%, most of which had healed by the end of the first postoperative year. These comparable healing rates reinforce the safety and biological viability of the subtrochanteric shortening osteotomy technique used in this cohort. When considered alongside the aforementioned studies, our data suggest that despite extensive soft-tissue release and the theoretically increased risk of vascular compromise, the proximal fragment maintains adequate perfusion to support timely bone healing and remodeling. This aligns with recent studies emphasizing the importance of controlled shortening and meticulous release for the preservation of vascular integrity. Delayed union, on the other hand, is more common and has been reported to correlate with the level of the osteotomy, the gap at the osteotomy site, and the length of femoral bone resection [27,29]. In the present study, nine patients (34.6%) were identified with delayed union at the sixth month visit. While the majority of these patients went on to heal by the end of the first year, there was one case (3.8%) of nonunion in whom revision is pending.

SPECT imaging combined with scintigraphy demonstrates the osteoblastic activity of the bones surrounding the prosthesis, and is less susceptible to metal-related artifacts than magnetic resonance imaging (MRI). Compared to standard radiography, which provides only structural information, and MRI, which primarily assesses soft tissues and bone marrow, SPECT/CT uniquely combines structural and functional data, allowing the objective and quantitative evaluation of bone perfusion following subtrochanteric osteotomy [4,5]. Although the scintigraphy component of SPECT/CT lacks detailed anatomical information, this limitation is compensated through the addition of CT imaging. The application of this approach in orthopedic imaging is still evolving, and so studies assessing its use for the evaluation of patients with total hip arthroplasty (THA) remain limited. Existing studies focus primarily on common postoperative complications such as aseptic loosening, periprosthetic infection, histiocytic reactions, periprosthetic fractures, polyethylene wear, and pseudo-tumor formation. To the best of our knowledge, there have been no studies to date analyzing changes in bone perfusion following THA in highly dislocated hips requiring extensive soft tissue release. A comparison of the SUV_mean_ values in the region between the greater and lesser trochanters, as well as tracer uptake ratios of the greater trochanter/sacrum and greater trochanter/distal femur in unilaterally operated patients in the present study revealed no statistically significant differences. These findings suggest that extensive soft tissue releases combined with subtrochanteric transverse shortening osteotomy in Crowe type III–IV DDH hips do not lead to significant changes in bone perfusion. Although theoretically, postoperative nonviable areas could result in decreased tracer uptake, only a small number of patients demonstrated hypoactivity on visual assessment in the study. The preservation of perfusion despite extensive soft-tissue release can be explained by the rich collateral vascular network of the proximal femur. The gluteal arteries, particularly the superior and inferior gluteal branches, together with the medial circumflex femoral artery, provide multiple anastomotic channels around the trochanteric region. Even when some periosteal and capsular vessels are disrupted during exposure and osteotomy, the metaphyseal and endosteal collateral flow likely maintain sufficient perfusion to prevent ischemia. Furthermore, the revascularization process following osteotomy may contribute to the normalization or even hyperactivity of the tracer uptake observed in SPECT/CT imaging during later follow-ups. Previous studies using SPECT/CT in asymptomatic patients following hip arthroplasty have reported increased tracer uptake around the greater trochanter and prosthesis beyond 12 months postoperatively, which is unrelated to aseptic or septic loosening. These findings are consistent with and support our results [30,31]. Clinically, preserved perfusion on SPECT/CT supports early mobilization and gradual weight-bearing after surgery. In revision cases, SPECT/CT may help identify well-perfused bone areas suitable for fixation or grafting.

Despite its findings, this study has several limitations, primarily its retrospective design and the relatively small number of patients. Given that Crowe type III and IV dislocated hips are relatively uncommon in routine clinical practice, we aimed to include all patients operated on at our center to minimize selection bias. Another limitation to be considered is the heterogeneous nature of the study cohort, which included both type III and IV dislocated hips. Furthermore, six patients underwent bilateral surgery, making comparisons with healthy contralateral hips difficult. The small sample size also precluded meaningful correlation or regression analyses, while the relatively short follow-up period limited the assessment of long-term outcomes. Finally, the absence of preoperative baseline perfusion data prevents any direct comparison of perfusion before and after surgery.

Future studies should adopt a prospective design and should include larger cohorts to validate the findings of the present study. Incorporating AI-assisted image analysis techniques could enhance the precision and reproducibility of perfusion quantification on SPECT/CT, allowing for automated segmentation and a more objective assessment of regional bone viability.

Finally, we used a SPECT/CT scan to evaluate perfusion of the proximal fragments of the osteotomy. While this has proven to be an effective method, concerns have been raised regarding the higher radiation exposure. In our study, the SPECT was hybridized with low-dose CT, ensuring that the total radiation exposure did not exceed 9 mSv. Previous studies in the literature investigating radiation exposure associated with SPECT/CT have reported that the total radiation exposure ranges between 8 and 10 mSv, which is equivalent to the dose of a thin-section pelvic CT scan [32–34].

## 6. Conclusion

In patients with Crowe types III–IV hip dysplasia undergoing total hip arthroplasty, shortening osteotomies and advanced soft tissue releases did not significantly reduce vascularity or perfusion in the proximal osteotomy fragment, as assessed by SPECT/CT. In our cohort, 61.5% of hips achieved radiographic union within 6 months, 34.6% of hips achieved radiographic union within 6–9 months (delayed union) and one case of nonunion were observed. These findings demonstrate that, despite advanced soft tissue releases, preserved proximal femoral perfusion correlates with high union rates, while careful follow-up remains essential for the detection and management of rare complications such as delayed union (34.6%) or nonunion (3.8%).

## Figures and Tables

**Table 1:** Demographic data of the study cohort.

	n=26
**Age**	
Mean ± SD	51.5 ± 8.8
Median (min–max)	51 (37–71)
**Sex**	
Male	5 (19.2%)
Female	21 (80.8%)
**Side**	
Right	14 (53.8%)
Left	12 (46.2%)
**Crowe type**	
III	5 (19.2%)
IV	21 (80.8%)
**Follow-up (months)**	
Mean ± SD	20.1 ± 10.1
Median (min–max)	18 (12–49)

**Table 2:** Union status and shortening amount of the operated hips.

	n=26
**Union type**	
Timely union (≤6 months)	16 (61.5%)
Nonunion	1 (3.8%)
Delayed union (6–9 months)	9 (34.6%)
**Time to union (n=25)**	
Mean ± SD	6.8 ± 1.7
Median (min–max)	6 (5–12)
**Final status at the end of the 1st year**	
Union	25 (96.2%)
Nonunion	1 (3.8%)
**Amount of shortening (cm)**	
Mean ± SD	3.8 ± 1.2
Median (min–max)	3.5 (2.0–7.0)

**Table 3:** SPECT/CT perfusion data of the operated and contralateral side (Tg/Sacrum: greater trochanter to sacrum tracer uptake ratio, Tg/Femur: greater trochanter to distal femur tracer uptake).

Parameter	Operated side (mean ± SD [95% CI])	Contralateral side (mean ± SD [95% CI])	p-value
**SUV** **_mean_**	3.5 ± 1.2 [2.97–4.03]	3.3 ± 1.2 [2.77–3.83]	0.350
**GT/Sacrum ratio**	0.6 ± 0.1 [0.56–0.64]	0.5 ± 0.1 [0.26–0.74]	0.425
**GT/Femur ratio**	1.4 ± 0.5 [1.18–1.62]	1.3 ± 0.4 [1.12–1.48]	0.674
